# Old T cells pollute with mito-litter

**DOI:** 10.1038/s43587-023-00412-2

**Published:** 2023-05-01

**Authors:** Manuel M. Gómez de las Heras, María Mittelbrunn

**Affiliations:** 1 Departamento de Biología Molecular, Facultad de Ciencias, Universidad Autónoma de Madrid, Madrid, Spain; 2 Homeostasis de Tejidos y Órganos, Centro de Biología Molecular Severo Ochoa, Consejo Superior de Investigaciones Científicas (CSIC) and Universidad Autónoma de Madrid, Madrid, Spain

## Abstract

The mysteries behind immune aging and its related inflammation are being unmasked. The research of Jin et al. reveals that the defective turnover of damaged mitochondria in CD4^+^ T cells from aged individuals results in the exacerbated secretion of mitochondrial DNA, fuelling inflammaging and impairing immune responses.

People make a big effort to recycle their waste products in order to prevent environmental pollution. Similarly, cells invest a lot of energy in recycling their components, however, any defect in the process could finish in littering their surroundings with molecular garbage. In this paper of *Nature Aging,* Jin and colleagues demonstrate a new cause-effect relationship between T cell aging and inflammaging. Their findings uncover that the intracellular degradation machinery of CD4^+^ T cells becomes faulty with age, so non-degraded products including damaged mitochondria and their DNA (mtDNA) are ultimately expelled to the extracellular environment fuelling inflammaging^1^. These results shed light on new molecular targets to ameliorate inflammaging in older people.

Inflammaging is referred to as a low-grade systemic inflammatory state in association with aging, characterised by high concentrations of proinflammatory mediators in serum (e.g., TNF and IL-6). Although it has been attributed to the accumulation of senescent cells, lifelong infections or the loss of gut barrier integrity, a series of recent articles place T cells as active contributors of inflammaging^2^. For example, the expansion of granzyme K-producing CD8^+^ T cells amplifies systemic inflammation and tissue senescence^3^, and mitochondrial and lysosomal stress in CD4^+^ and CD8^+^ T cells results in premature inflammaging that accelerates organismal senescence and aging in mice^4,5^. In support of T cells as mediators of inflammaging, this remarkable new paper demonstrates that aged CD4^+^ T cells with a defective autophagy system drive inflammaging by the secretion of non-degraded damage products to the extracellular medium.

Cells harbour a variety of mechanisms that detect, mark and eliminate intracellular molecules and organelles that are damaged or need to be recycled to ensure homeostasis. For instance, the proteasome pathway breaks down ubiquitin-labelled proteins into small peptides, and the autophagy system is responsible for the turnover of intracellular components, such as mitochondria, making use of the endolysosomal compartment to degrade them. Thereby, injured organelles are sealed into autophagosomes, which require processing and fusion with lysosomes where luminal contents are degraded in an acidic environment.

This research by Jin et al. indicates the ubiquitin-proteasome and autophagy pathways regulate each other and both are altered in old T cells. They describe that the age-associated decline in TCF1, a transcription factor related to T-cell stemness^6^, enhances the expression of the gene encoding for the cytokine-inducible SH2-containing protein (CISH). The study illustrates that CISH binds directly to ATP6V1A, a catalytic subunit of the lysosomal proton pump ATPase complex, favouring its ubiquitination and subsequent proteasomal degradation. Consequently, lysosomal acidification is diminished and, thus, its recycling function (Figure 1). Therefore, the age-related upregulation of CISH in T cells leads to lysosome dysfunction.

This paper reveals that the age-associated blockade of autophagy flux in human CD4^+^ T cells expands the entire endolysosomal compartment, including the accumulation of multivesicular bodies (MVBs) containing exosomes, autophagosomes, amphisomes and autolysosomes. However, their luminal contents are not degraded fostering the intracellular accumulation of damaged components. Bektas et al. previously reported that CD4^+^ T cells from aged individuals exhibited an increased number of dysfunctional mitochondria in the autophagic compartment, reflecting a defect in mitochondrial recycling^7^. Accordingly, Jin and colleagues observe that autophagy-impaired human CD4^+^ T cells accumulate amphisomes charged with damaged mitochondria during aging (Figure 1), depicting a new molecular mechanism by which lysosomal function is corrupted during T cell aging.

Then, how is this molecular garbage managed when the recycling machinery does not work properly? Jin et at. describe in their current paper of *Nature Aging* that aged CD4^+^ T cells secrete amphisome-derived exosomes together with damaged mitochondria components, increasing the concentration of extracellular mtDNA (Figure 1). These findings fit with previously published data from these authors showing that lysosomal dysfunction in CD4^+^ T cells from old individuals prompted the secretion of granzyme B-enriched exosomes with highly cytotoxic properties to the neighbourhood cells^8^. In addition, circulating levels of mtDNA in humans augment with age in parallel with the concentration of proinflammatory cytokines^9^ and, strikingly, Jin and colleagues correlate the levels of T cell-derived mtDNA with parameters of inflammaging. Adoptively transferring antigen-specific CD4^+^ T cells to young immunized mice increase serum levels of mtDNA along with the concentration of TNF and IL-6, which is prevented by silencing *CISH* in donor T cells.

Mechanistically, it is known that mtDNA is sensed as a damage-associated molecular pattern (DAMP) through the endosomal TLR9, or via the cytosolic cGAS/STING and NLRP3/inflammasome pathways, all converging on the activation of a proinflammatory program^10^. Whether mtDNA derived from old CD4^+^ T cells is secreted naked or associated with exosomes or other types of vesicles, such as mitochondria-derived vesicles^11^, in this scenario requires further investigation. CD4^+^ T cells are able to transfer mtDNA via exosomes to nearby immune cells activating their intracellular cGAS/STING pathway^12^. Thereby, mtDNA from damaged mitochondria could be shuttled from old CD4^+^ T cells to the extracellular medium and reach other bystander cells firing the aforementioned inflammatory signalling cascades. However, it is still opened how secreted mtDNA from aged T cells is sensed by surrounding cells provoking inflammaging. Importantly, the extracellular release of other mitochondria-derived DAMPs (i.e., cardiolipin, N-formyl peptides, ATP or Tfam) could also act as immunomodulatory cues in the development of inflammaging^10^.

Inflammaging underlies also defective immune responses of older people, who have a higher susceptibility to infectious and oncologic diseases as well as a poor vaccination efficacy. Surprisingly, targeting the CISH-induced lysosomal dysfunction in CD4^+^ T cells not only attenuates premature inflammaging, but also improves antibody responses in young recipient mice followed by a viral and non-infectious challenge. In particular, immunized mice receiving CISH-deficient CD4^+^ T cells display an increased number of T follicular cells, which tailor T-dependent antibody responses and, accordingly, an increased production of antigen-specific antibodies. Recent findings have uncovered that knocking-out CISH enhances T cell anti-tumor activity and susceptibility to PD-1 blockade, being the foundation of a current human clinical trial testing adoptive T cell therapy to treat gastrointestinal cancer patients (ClinicalTrials.gov Identifier NCT04426669)^13^. Interestingly, Jin et al. results suggest that *CISH* silencing could mitigate complications derived from adoptive T cell therapy such as inflammatory cytokine release syndrome, an important challenge in cancer immunotherapy. Therefore, this novel study could have important clinical implications with the aim to boost T-cell immunity while keeping inflammation at bay.

This timely piece of work reinforces the idea that old T cells with defective mitochondria have an active role in inflammaging. The defective disposal of dysfunctional mitochondria through autophagy in CD4^+^ T cells from aged individuals results in the extracellular secretion of mtDNA fuelling chronic inflammation. This research not only supports the growing body of evidence showing an age-dependent decline in the CD4^+^ T cell autophagy system^7,8,14^, but also highlights its relevance in mitochondrial quality control, providing mechanistic insights into how old T cells accumulate dysfunctional mitochondria. Interestingly, mimicking the age-related mitochondrial decline in T cells results in lysosome dysfunction and alterations in the autophagic flux^4,5^. Thereby, the intimate bidirectional crosstalk between the endolysosomal system and mitochondria could be exploited to rejuvenate the T cell compartment as well as to put off inflammaging to foster healthier aging.

## Figures and Tables

**Figure 1:**
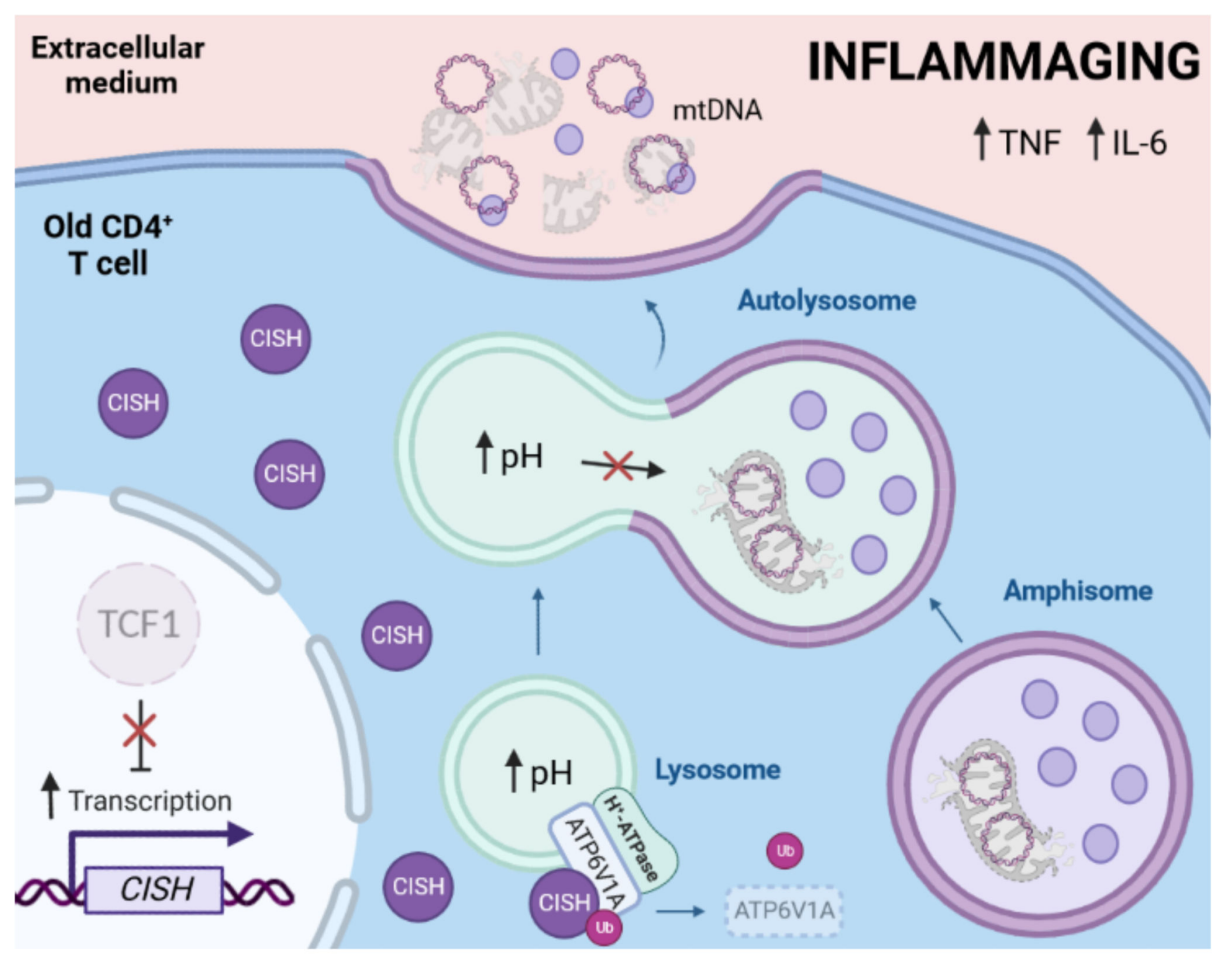
Lysosomal dysfunction in T cell aging fosters mtDNA secretion and inflammaging. The age-dependent decline in TCF1 in human CD4^+^ T cells upregulates *CISH* gene transcription, which encodes for a scaffolding protein involved in protein ubiquitination. CISH binds and facilitates the ubiquitin-dependent degradation of ATP6V1A, a catalytic module of the lysosomal proton (H^+^) pump ATPase, leading to lysosomal dysfunction. Consequently, there is an accumulation of non-degraded cargos including exosomes and dysfunctional mitochondria in the endolysosomal system (i.e., amphisomes), which are ultimately released to the extracellular milieu serving as source of mitochondrial DNA (mtDNA) and correlating with inflammaging. mtDNA: mitochondrial DNA; Ub: ubiquitin.
